# Prevalence of Trachoma in Benishangul Gumuz Region, Ethiopia, after Implementation of the SAFE Strategy: Results of Four Population-Based Surveys

**DOI:** 10.1080/09286586.2022.2140439

**Published:** 2022-12-13

**Authors:** Belete Mengistu, Fikru Wirtu, Addisu Alemayehu, Shigute Alene, Aemiro Asmare, Sharone Backers, Ana Bakhtiari, Molly Brady, Robert M. R. Butcher, Mihiret Dayessa, Hannah Frawley, Genet Gebru, Cristina Jimenez, Fikreab Kebede, Asfaw Kejela, Scott McPherson, Addisalem Mihret, Nebiyu Negussu, Jeremiah M. Ngondi, Fentahun Taddese, Rebecca Willis, Asfaw Wondimu, Michael Dejene, Anthony W. Solomon, Emma M. Harding-Esch

**Affiliations:** a Act to End NTDs East, RTI International, Addis Ababa, Ethiopia; b Health Promotion and Disease Prevention Core Process, Benishangul-Gumuz Regional Health Bureau, Ethiopia; c Task Force for Global Health, Atlanta, Georgia, USA; d Act to End NTDs East, RTI International, Washington, DC, USA; e Clinical Research Department, London School of Hygiene & Tropical Medicine, London, UK; f Ambo Hospital, Ambo, Ethiopia; g Neglected Tropical Diseases Team, Disease Prevention and Control Directorate, Ministry of Health, Ethiopia; h Sightsavers, Haywards Heath, UK; i Asfaw Wondimu Health Research and Consultancy, Addis Ababa, Ethiopia; j Sightsavers, Addis Ababa, Ethiopia; k Department of Control of Neglected Tropical Diseases, World Health Organization, Geneva, Switzerland

**Keywords:** Trachoma, Ethiopia, Benishangul Gumuz, neglected tropical diseases, elimination, prevalence

## Abstract

**Purpose:**

We aimed to estimate the prevalence of trachomatous inflammation–follicular (TF) in 1–9-year-olds and trachomatous trichiasis (TT) unknown to the health system in ≥15-year-olds in Benishangul Gumuz (BGZ) region, Ethiopia. This will help to assess progress towards the elimination of trachoma as a public health problem and determine the need for future interventions against trachoma in the region.

**Methods:**

Cross-sectional population-based trachoma prevalence surveys were conducted in four evaluation units (EUs) of BGZ using World Health Organization-recommended survey methodologies. Individuals were examined for clinical signs of trachoma. Household access to water, sanitation and hygiene facilities (WaSH) was assessed.

**Results:**

A total of 11,778 people aged ≥1 year were examined. The prevalence of TF in 1–9-year-olds was <5% in three EUs and ≥5% in one EU. The prevalence of TT unknown to the health system in people aged ≥15-years was ≥0.2% in all four EUs. The proportion of households with an improved drinking water source within a 30-minute round-trip ranged from 27−60%. The proportion of households with an improved latrine ranged from <1−6%.

**Conclusions:**

Surgical interventions for TT are required in all EUs in BGZ. One annual round of mass drug administration (MDA) of azithromycin is required in one EU before resurvey to reassess progress in lowering TF prevalence below the WHO elimination threshold of 5% in 1–9-year-olds. MDA should be stopped in the other three EUs and trachoma surveillance surveys should be conducted at least 24 months after the surveys described here. Ongoing strengthening of WaSH infrastructure may help sustain the low prevalence of trachoma.

## Introduction

Trachoma is an infectious disease of the eye caused by the bacterium *Chlamydia trachomatis*.^1^ Repeated infection^2^ and the associated inflammatory response lead to scarring of the tarsal conjunctivae, trachomatous trichiasis (TT), corneal opacity and visual impairment.^1^ Infection spreads from person-to-person on hands, fomites and flies that have been in contact with discharge from the eyes or nose of an infected person.^3–5^ Trachoma is more common in people who live in poor and overcrowded communities with lack of access to water and sanitation.^6–9^ In May 2020, globally, a total of 137 million people were living in endemic areas and of these, 50% (68.5 million) were in Ethiopia.^10^

Trachoma can be eliminated as a public health problem through the use of a package of interventions known as the Surgery, Antibiotics, Facial cleanliness and Environmental improvement (SAFE) strategy.^11,12^ Elimination is achieved when the prevalence of TT unknown to the health system is <0.2% among people aged ≥15 years, prevalence of trachomatous inflammation–follicular (TF) is <5% in children aged 1–9 years, and there is written evidence of a system to identify and manage incident cases of TT, in all formerly endemic districts.^13^

Between December 2013 and January 2014, the Benishangul Gumuz (BGZ) Regional Health Bureau participated in the Global Trachoma Mapping Project (GTMP), undertaking baseline surveys to determine the need for implementation of the SAFE strategy in the region. The 20 woredas (districts) in the region were grouped into seven evaluation units (EUs), based on common borders and similar socioeconomic characteristics. The prevalence of TT in people aged ≥15 years was ≥0.2% in all seven surveyed EUs.^14^ Four EUs (covering 11 woredas) were identified in which the prevalence of TF was ≥5% and therefore qualified for implementation of the A, F and E components of SAFE. Among these four EUs, TF prevalence was ≥10% in two EUs covering four woredas (Pawe, Mandura, Bulen and Dibate) which, therefore, required three rounds of annual antibiotic mass drug administration (MDA). The other two EUs in which interventions were required, covering seven woredas (Homesha, Sherkole, Menge, Kurmuk, Dangur, Wembera, and Guba), had TF prevalences between 5 and 9.9% and were eligible to receive one round of MDA.^14^

As a result of the GTMP survey data, MDA was conducted in all four EUs with TF prevalence ≥5%. The two EUs (four woredas) requiring three rounds started MDA in 2014 just after the baseline surveys finished; one round of MDA was delivered to the remaining two EUs (seven woredas) in 2017. Active TT case finding and corrective surgery were launched throughout the region in 2019 as well as implementation of water, sanitation and hygiene (WaSH) interventions and coordinated neglected tropical disease activities targeting trachoma.^15,16^

This survey series aimed to determine how far BGZ remains from elimination of trachoma as a public health problem. We used population-based trachoma surveys to measure prevalence of TT unknown to the health system and TF after the interventions undertaken so far, to provide evidence with which to plan whether future interventions are needed.

## Methods

### Ethical approval and consent

Ethical approval was obtained from the ethics committees of the BGZ Regional Health Bureau and the London School of Hygiene & Tropical Medicine (16105). Regional, zonal and woreda officials were engaged and provided letters of support. The purpose of the survey was explained to the members of each participating household before enrollment and informed verbal consent was obtained from all adult participants before examination. For participants aged <18 years, informed verbal consent was obtained from the head of household, a parent or guardian. Consent was documented electronically using the Tropical Data app (https://www.tropicaldata.org/). All individuals noted to have active trachoma were provided with 1% tetracycline eye ointment, and those with TT were referred to the nearest appropriate healthcare facility for surgical correction.

### Study setting

**Figure 1.**
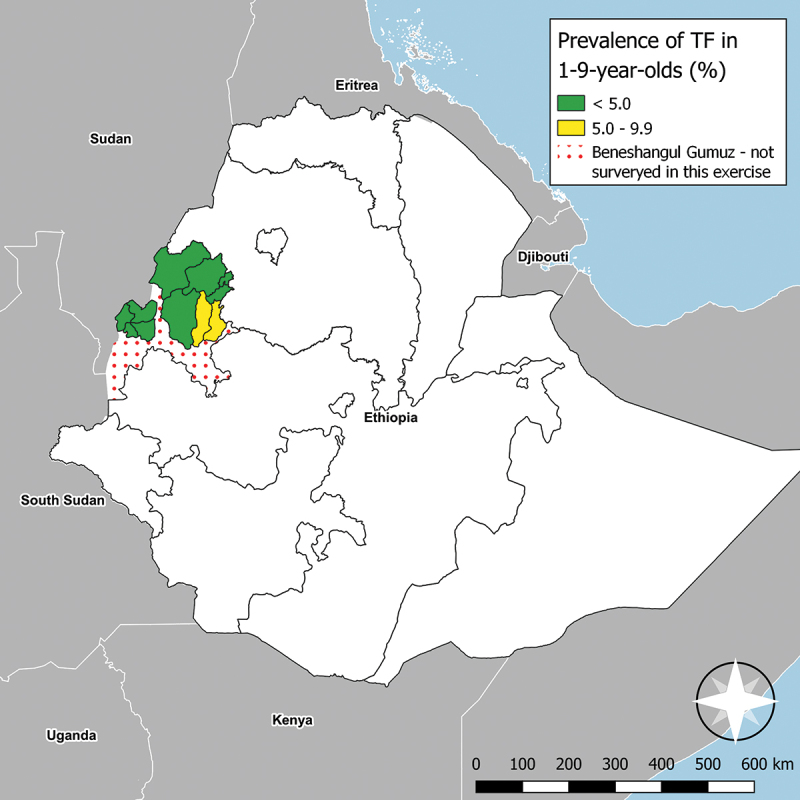
Prevalence of trachomatous inflammation–follicular (TF) measured at trachoma impact surveys in Benishangul Gumuz, Ethiopia, March 2018− March 2019. The prevalence of trachomatous trichiasis unknown to the health system measured simultaneously in all four EUs shown here was between 0.4 and 0.9%. The boundaries and names shown and the designations used on this map do not imply the expression of any opinion whatsoever on the part of the authors, or the institutions with which they are affiliated, concerning the legal status of any country, territory, city or area or of its authorities, or concerning the delimitation of its frontiers or boundaries.

BGZ is one of the eleven regional states of Ethiopia (Figure 1, map). It is located in the south west of the country, with the 2017 population estimated to be 1,066,001.^17^ The region is divided into one town and three zones, currently sub-divided into 20 woredas containing a total of 475 kebeles (the smallest administrative units for which population estimates are available). For these trachoma impact surveys, four EUs were created with boundaries that matched those from the pre-MDA baseline surveys.^14^

### Study design and participant selection

A two-stage cluster sampling design was developed that complied with World Health Organization (WHO)-recommended technical parameters for trachoma surveys.^18^ The sample size was calculated to estimate a prevalence of TF in 1−9-year-olds of 4% with an absolute precision of ±2% at the 95% confidence level, assuming a design effect of 2.63^18,19^ and accounting for non-response with an inflation factor of 1.2. TT was determined using the number of people aged ≥15 years living in selected households.^20^

In the first stage, villages (clusters) were sampled. In each EU, a complete list of villages was obtained from the BGZ Regional Health Bureau, and 26 were selected systematically with a probability of selection proportional to population size. In the second stage, 30 households were selected per cluster using compact segment sampling. All residents of selected households aged ≥1 year were eligible for inclusion.^20^

### Training

The training for both graders and recorders was conducted over four days using version 2 of the Tropical Data standardized training programme.^21^ For trainee graders to be certified for deployment to the field for data collection, they had to pass two inter-grader agreement assessments, comparing their grades to expert consensus on a set of photographs, followed by a comparison to a certified grader trainer on 50 live participants. Only graders who achieved a kappa of ≥0.7 for TF on both assessments participated in the survey. Survey recorders were trained to recognize water, sanitation and hygiene facility types, and to utilize the Tropical Data electronic data capture system. The recorder had to attain an agreement of 90% on a set of test data to be certified. Qualified grader and recorder pairs participated in practical team training before beginning field work.

### Clinical assessment

Both eyes of all consenting residents aged ≥1 year were examined using adequate light (torch or sunlight) and a 2.5× magnifying loupe. Participants were examined for the presence of TT, TF and trachomatous inflammation–intense (TI) using the WHO simplified trachoma grading system.^22^ For individuals who had TT, additional questions were asked about whether they had been offered and/or taken up management for TT, and the conjunctiva was examined for trachomatous scarring (TS). TT “unknown to the health system” was defined as an eye with TT for which the participant could not remember being offered surgery or epilation.

### Water, sanitation and hygiene data collection

Information on WaSH access was collected by recorders through interviews with heads of households and observation of sanitation and handwashing facilities. Variables related to water access and source type for both drinking and washing, and sanitation types were collected using a version of the WHO/United Nations Children’s Fund (UNICEF) Joint Monitoring Programme (JMP) household questionnaire adapted for the context of trachoma.^20^

### Data quality control, quality assurance and analysis

Data quality measures were implemented as previously described.^23,24^ Descriptive data and prevalence results were analyzed using open source R-based analysis scripts (https://github.com/itidat/tropical-data-analysis-public), as described elsewhere.^20^ TF prevalence in children aged 1–9 years was adjusted for age in 1-year age-bands, and TT prevalence in people aged ≥15 years was adjusted for age and gender in 5-year age-bands, using 2007 census data.^25^ Confidence intervals (CIs) were produced by bootstrap resampling of adjusted cluster-level outcome proportions.^26^ WaSH facilities were categorized according to the WHO/UNICEF JMP definitions.^27^Associations between TF and individual- and household-level variables were analyzed using mixed-effects binomial regression, in a similar approach to that of previous studies.^28^ As there were only four EUs surveyed in this series, the number of levels was insufficient to warrant inclusion of EU as a random effect. Similarly, the number of TF cases was insufficient to enable convergence of complex multivariable models with household included as a random effect. Cluster of residence was, therefore, the only random-effect variable included. Age and gender were included *a priori* as fixed-effect variables due to their known relationship with trachoma. Household-level fixed-effect variables were first tested for correlation and removed or amalgamated where correlation was found based on a Spearman’s rho >0.8. Fixed-effect variables were then tested in univariable analysis; those for which evidence of association with TF was strong (p < .05) were carried into the multivariable analysis. Finally, stepwise addition and removal of further fixed-effect variables was undertaken to finalize model structure. Comparison between models was achieved through likelihood ratio testing (LRT), with and without the variable in question (involving comparison to a null model in the case of univariable analyses).

Associations with TT were analyzed using the same approach. All TT cases, regardless of management status, were included in this analysis.

## Results

### Study population

A total of four EUs (comprising 11 woredas) were surveyed from March 2018− March 2019. A total of 3,081 households were visited in 103 clusters, from which 13,100 people were enumerated. Of these individuals, 11,778 (89.9%) consented to participate and were examined, 1287 (9.8%) were absent at the time of the team’s visit and 0.3% refused to take part. Of those examined, 6,329 (53.7%) were female. A total of 4,590 children aged 1–9 years and 5,825 people aged ≥15 years were examined (Tables 1–3).

**Table 1.** Population aged ≥1 year participating in trachoma impact surveys in Benishangul Gumuz, Ethiopia, March 2018− March 2019.

Evaluation unit (EU)	EU ID	Enumerated	Absent	Refused	Other	Examined	Examined female (%)
Sherkole, Menge, Kurmuke & Homosha	80635	3607	315	9	0	3283	56
Wombera, Guba & Dangure	80636	2983	177	3	0	2803	53
Dibate & Bullen	80981	3267	416	9	0	2842	53
Pawe & Mandura	80982	3243	379	14	0	2850	52

**Table 2.** Children aged 1 − 9 years with trachomatous inflammation–follicular (TF) and trachomatous inflammation–intense (TI) in Benishangul Gumuz, Ethiopia, March 2018− March 2019.

Evaluation unit	Children aged 1–9 years examined	Children aged 1–9 years with TF	Children aged 1–9 years with TI	Age-adjusted prevalence of TF in 1–9 year olds (%, 95% CI)
Sherkole, Menge, Kurmuke and Homosha	1,375	30	0	2.18 (1.23–2.87)
Wombera, Guba and Dangure	1,030	56	5	4.71 (1.82–7.52)
Dibate and Bullen	1,157	117	10	9.06 (5.78–12.82)
Pawe and Mandura	1,028	56	5	3.98 (1.61–7.27)

CI: confidence interval.

**Table 3.** People aged ≥15 years with trachomatous trichiasis (TT) identified in trachoma impact surveys in Benishangul Gumuz, Ethiopia, March 2018− March 2019.

Evaluation unit	Adults aged ≥15 years examined	Adults aged ≥15 years with TT	Adults aged ≥15 years with TT unknown to the health system	Age- and gender-adjusted prevalence of TT unknown to the health system in ≥15-year-olds (%, 95% CI)
Sherkole, Menge, Kurmuke and Homosha	1472	27	25	0.93 (0.42 − 1.61)
Wombera, Guba and Dangure	1439	12	12	0.40 (0.14 − 0.77)
Dibate and Bullen	1411	15	15	0.68 (0.27 − 1.28)
Pawe and Mandura	1503	24	21	0.76 (0.46 − 1.16)

CI: confidence interval

#### Trachoma prevalence

TF was present in 259 (5.6%) of examined 1–9-year-olds. EU-level age-adjusted TF prevalence ranged from 2.2% (95% confidence interval [CI]: 1.2 − 2.9%) in Sherkole, Menge, Kurmuke and Homosha woreda of Assosa zone to 9.1% (95% CI: 5.8 − 12.8%) in Dibate and Bullen of Metekel Zone. There was only one EU which had a TF prevalence ≥5% (Figure 1, Table 2).

**Figure 2.**
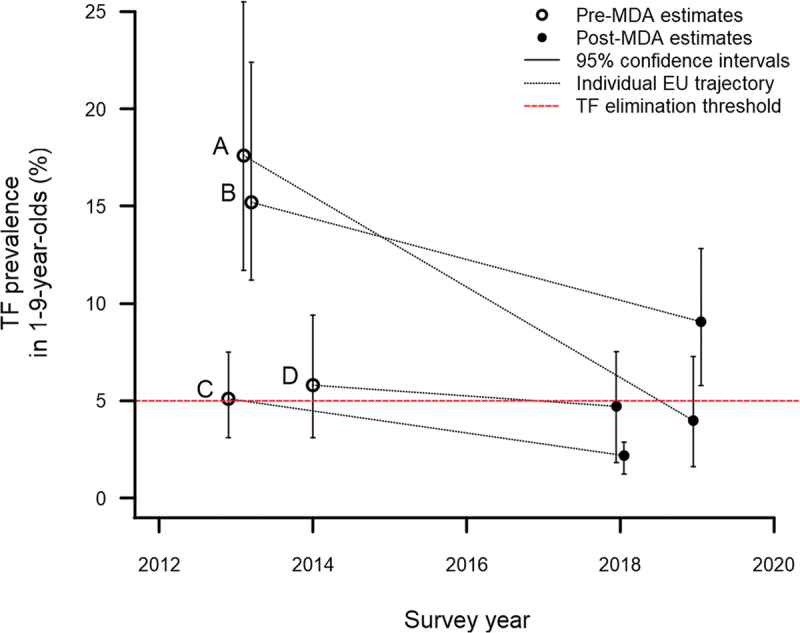
Change in trachomatous inflammation–follicular (TF) prevalence in 1–9-year-olds between baseline estimates (published elsewhere)^14^ and trachoma impact survey estimates March 2018− March 2019, Benishangul Gumuz, Ethiopia. (A) Pawe and Mandura; (B) Dibate and Bullen; (C) Sherkole, Menge, Kurmuke & Homosha; (D) Wombera, Guba & Dangure. X-coordinates have been artificially offset to allow the confidence interval whiskers to be clearly seen.

The change in prevalence from baseline to these impact surveys is shown in Figure 2. In three EUs, the post-MDA prevalence was <5%, suggesting MDA should be stopped and trachoma surveillance surveys undertaken after 2 years. In one EU, the TF prevalence decreased from 15.2% to 9.1% (Figure 2).

Overall, 78 cases of TT were identified across all EUs, of whom 73 (94%) reported not having been offered surgery or epilation and were classified as “unknown to the health system”. The age- and gender-adjusted prevalence of TT unknown to the health system in people aged ≥15 years ranged from 0.4% (95% CI: 0.1 − 0.8%) in Wombera, Guba and Dangure woredas to 0.9% (95% CI: 0.4 − 1.6%) in Sherkole, Menge, Kurmuke and Homosha woredas. The TT prevalence unknown to the health system in people aged ≥15 years was, therefore, ≥0.2% in all four EUs (Table 3).

#### Water, sanitation and hygiene access

The proportion of households with an improved drinking water source within a 30-minute round-trip ranged from 27 − 60%. The proportion of households with an improved latrine ranged from <1%−6%. The proportion of households with a latrine (status not specified) that had a hand wash station with water and soap was <1%−16% (Table 4).

**Table 4.** Water, sanitation and hygiene access of households in Benishangul Gumuz, Ethiopia, March 2018− March 2019.

Evaluation unit	Clusters	Households	Households with an improved drinking water source within 30-minute round trip (%)	Households with an improved latrine (%)	Households with a latrine with a hand wash station (%)
Sherkole, Menge, Kurmuke and Homosha	28	836	27	<1	7
Wombera, Guba and Dangure	23	680	31	4	<1
Dibate and Bullen	26	779	43	3	12
Pawe and Mandura	26	786	60	6	16

**Table 5.** Univariable and multivariable analysis of factors associated with trachomatous inflammation–follicular (TF) in children aged 1–9 years in trachoma impact surveys in Benishangul Gumuz, Ethiopia, March 2018─March 2019. Models all include cluster of residence as a random-effects variable to account for clustering in the survey data.

Variable	Levels	No TF	TF	Univariable analysis		Multivariable analysis	
				OR (95% CI)	P*	aOR (95% CI)	P†
Age group (years)	1–3	1,379	154	Ref	<0.001	Ref	<0.001
	4–6	1,540	88	0.49 (0.37–0.65)		0.48 (0.36–0.64)	
	7–9	1,412	17	0.11 (0.06–0.17)		0.10 (0.06–0.17)	
Gender	Male	2,259	122	Ref	0.235	Ref	0.064
	Female	2,072	137	1.17 (0.91–1.51)		1.29 (0.99–1.69)	
Washing water source status	Improved	3,211	203	Ref	0.133	Not tested	
	Unimproved	497	31	0.83 (0.46–1.49)			
	Surface	497	25	0.63 (0.39–1.00)			
Round trip to washing water source	<30 minutes	2,310	118	Ref	0.217	Not tested	
	≥30 minutes	2,021	141	1.22 (0.89–1.68)			
Latrine ownership	Private	2,587	88	Ref	<0.001	Ref	0.293
	Shared	447	14	1.17 (0.65–2.12)		1.11 (0.61–2.03)	
	Open	1,297	157	3.39 (2.41–4.79)		4.47 (0.79–25.4)	
Latrine status	Improved	89	3	Ref	<0.001	Ref	0.929
	Unimproved	1289	156	1.11 (0.33–3.68)		1.19 (0.35–4.04)	
	Open	2953	100	3.55 (1.06–11.89)		0.92 (0.11–7.62)	
Number of children aged 1–9 years living in the household	1–2	2,131	115	Ref	0.531	Not tested	
	3–4	1,970	127	1.11 (0.85–1.46)			
	≥5	230	17	0.85 (0.48–1.49)			

OR: odds ratio; aOR: adjusted odds ratio; CI: confidence interval; P: p-value

* Likelihood ratio test between univariable model and null model

† Likelihood ratio test between models with and without the variable in question.

#### Factors associated with trachoma

**Figure 3.**
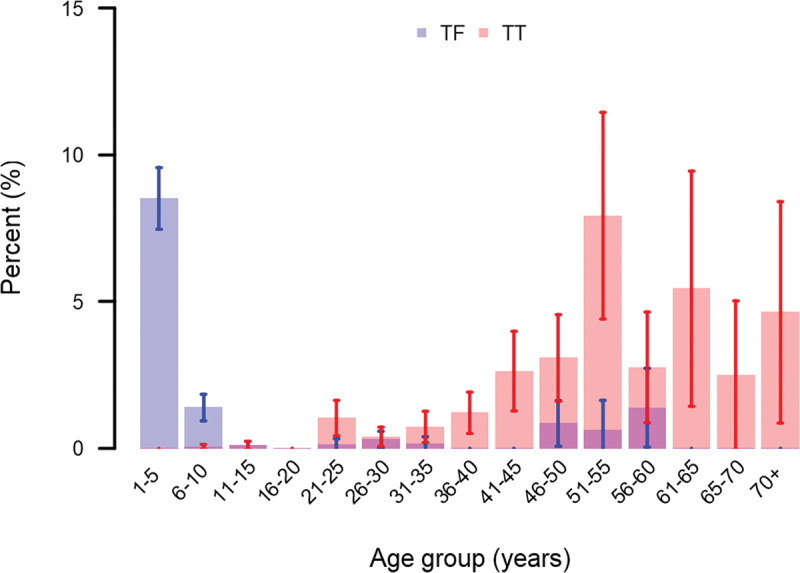
Age distribution of trachomatous inflammation–follicular (TF) and trachomatous trichiasis (TT; management status not specified) during trachoma impact surveys in four evaluation units (11 woredas) of Benishangul Gumuz, Ethiopia, March 2018─March 2019. Whiskers represent 95% confidence intervals around age-specific proportions.

Compared to 1–3-year-olds, TF was less common in 4–6-year-olds (adjusted odds ratio [aOR]: 0.48, 95% CI: 0.36─0.64) and still less common in 7–9-year-olds (aOR: 0.10, 95% CI: 0.06─0.17; p < .001). TF tended towards being more common in female than male children; however, there was weak evidence to reject the null hypothesis of no gender difference (aOR1.29, 95% CI: 0.99─1.69; p = .064). None of the household-level WaSH variables measured were associated with TF (Table 5). The age-distribution of TF in five-year age brackets is shown in Figure 3.

TT was more common in older individuals and in females. Compared to the reference age group of 15–35 years, the aOR for TT in 36–55-year-olds was 6.6 (95% CI: 3.8–11.5), for 56–75-year-olds was 8.5 (95% CI: 4.20–17.9) and for >75-year-olds was 18.0 (95% CI: 5.8–56.1; LRT: p < .001). The aOR for TT in females compared to males was 3.9 (95% CI: 2.2–7.0; LRT: p < .001). The age-distribution of TT in five-year age brackets is shown in Figure 3.

## Discussion

In BGZ, the prevalence of TF in 1–9-year-olds was lower following implementation of MDA than at baseline. In three of four EUs surveyed, this reduction allowed MDA to be discontinued. This is encouraging, given the importance of Ethiopia to the global trachoma program overall.^29^ In the fourth EU surveyed, a substantial decrease in TF prevalence was observed, but one additional round of MDA is still required before another impact survey can be conducted, as per WHO recommendations.^30^

This survey series was not designed to measure changes in TT prevalence between the pre-MDA and post-MDA surveys: the sample did now allow generation of sufficient precision in TT prevalence estimates. However, it is notable that the prevalence of TT remained above the elimination threshold (a prevalence of TT “unknown to the health system” <0.2% in adults aged ≥15 years: approximately 1 case per 1000 total population) in all four EUs, just as it was during the GTMP surveys several years previously. With 94% of individuals with TT reporting not having been offered management surgery or epilation, increased TT surgery provision and uptake are urgently needed. Active TT case finding should take place and surgical services should continue to be strengthened. The age and gender distribution of TT suggest programs should ensure older women, in particular, have equitable access to TT management.

WaSH access in BGZ also requires improvement. Between one- and two-thirds of all households in the areas we surveyed did not have access to a water source within a 30-minute round trip. This could lead to prioritization of water for drinking and cooking and, thus, limited water use for hygiene purposes, particularly (from a trachoma perspective) facial cleanliness. An overwhelming majority of households did not have an improved sanitation facility. Unimproved latrines and open defecation could serve as breeding sites for the flies which transmit ocular *C. trachomatis*.^3,31^ Guidelines for sustainable elimination of NTDs, including trachoma, suggest that prolonged investment in WaSH infrastructure beyond meeting disease elimination thresholds would be needed to maintain the benefits of MDA programmes.^32^ This is perhaps beyond the scope of the trachoma programme alone. Instead, these data could add leverage to the case for ongoing, general improvements to WaSH infrastructure.

One limitation of this study was the modelling approach used to identify factors potentially associated with TF. Pre-filtering variables for inclusion in a multivariable model risks falsely reducing variance and over-fitting the model. In this case, we elected to pre-filter the variables on the basis that the variables we had collected data on would not cumulatively predict trachoma risk. Instead, our aim was to estimate the magnitude of any relationship between household WaSH facility access and TF, for which an explanatory model would be preferable, and inclusion of unrelated variables could confound the estimate. Despite the risk of overfitting, no significant relationships were identified between TF and WaSH variables. The lack of association between TF and household-level WaSH variables should not deter regional health authorities from greater focus on the F and E components of SAFE. Because of the relatively low current prevalence of TF in these EUs, analysis of the small number of TF cases was probably underpowered to detect associations of TF and WaSH variables. Larger, multi-country analyses provide compelling evidence of association.^8^

These data represent a significant step forwards towards elimination of trachoma in BGZ. We have demonstrated that, in addition to the woredas found to have a TF prevalence <5% at the time of the GTMP, a further nine woredas no longer require MDA. Program momentum should be maintained to ensure the gains made by MDA are sustained long-term.
